# Long-term culture of feline oviduct epithelial cells on permeable filter supports

**DOI:** 10.1007/s10616-022-00542-2

**Published:** 2022-09-05

**Authors:** Susanne Eder, Karin Müller, Shuai Chen, Jennifer Schoen

**Affiliations:** 1 Department of Reproduction Biology, Leibniz Institute for Zoo and Wildlife Research, Berlin, Germany; 2 Institute of Reproductive Biology, Institute for Farm Animal Biology, Dummerstorf, Germany

**Keywords:** Oviduct epithelial cells, Long-term culture, Domestic cat

## Abstract

Basic knowledge about cellular and molecular mechanisms underlying feline reproduction is required to improve reproductive biotechnologies in endangered felids. Commonly, the domestic cat (*Felis catus*) is used as a model species, but many of the fine-tuned, dynamic reproductive processes can hardly be observed in vivo. This necessitates the development of in vitro models. The oviduct is a central reproductive organ hosting fertilization in the ampulla and early embryonic development in the isthmus part, which also functions as a sperm reservoir before fertilization. In other species, culturing oviduct epithelial cells in compartmentalized culture systems has proven useful to maintain oviduct epithelium polarization and functionality. Therefore, we made the first attempt to establish a compartmentalized long-term culture system of feline oviduct epithelial cells from both ampulla and isthmus. Cells were isolated from tissue samples (n = 33 animals) after routine gonadectomy, seeded on permeable filter supports and cultured at the liquid–liquid or air–liquid interface. Cultures were harvested after 21 days and microscopically evaluated for epithelial differentiation (monolayer formation with basal–apical polarization) and protein expression of marker genes (oviduct-specific glycoprotein, acetylated tubulin). Due to the heterogeneous and undefined native tissue material available for this study, the applied cell culture approach was only successful in a limited number of cases (five differentiated cultures). Even though the protocol needs optimization, our study showed that the compartmentalized culture approach is suitable for maintaining differentiated epithelial cells from both isthmus and ampulla of the feline oviduct.

## Introduction

Many felid species are classified as near threatened or threatened on The IUCN Red List of Threatened Species (https://www.iucnredlist.org/en). To preserve these species and their genetic diversity, the continuous improvement of assisted reproductive techniques (ARTs) based on a better understanding of feline reproduction is urgently needed.

The oviduct and especially the epithelium lining the oviduct lumen creates the optimal milieu for central reproduction events such as final gamete maturation, fertilization, and early embryo development (reviewed in: Coy et al. 2012). However, to date there is only little knowledge about feline oviduct epithelium physiology. As in vivo experiments are technically challenging and ethically questionable, in vitro models are established in accordance to the 3R principle (replacement, refinement, reduction of animal experiments). So far, mainly suspension cultures (Lengwinat et al. 1992; Eder et al. 2020) or in one case, 2D adherent submerged cultures (Roth et al. 1993) have been used for studying feline oviduct epithelium physiology. The two main applications of feline oviduct epithelial cell (FOEC) cultures were the characterization of sperm–oviduct interactions (Henry et al. 2015; Eder et al. 2020) and the improvement of fertilization and developmental competence of early cat embryos (Lengwinat et al. 1992; Roth et al. 1993; Swanson et al. 1996). However, studies on bovine and porcine oviduct epithelial cells have shown that 2D adherent submerged and suspension culture conditions rapidly lead to changes in cell type-specific morphology and functionality, including loss of polarization, impaired barrier formation, and altered gene expression (Reischl et al. 1999; Rottmayer et al. 2006; Danesh Mesgaran et al. 2016; Chen and Schoen 2019). Therefore, they are not applicable for long-term functional studies.

In compartmentalized culture systems, cells grow on permeable filter supports (inserts) hanging or standing in the cell culture dish. Medium is provided from below the filter support. This culture approach is preferentially used for epithelial cells as it ensures in vivo-like nutrition via the basal cell side and triggers the formation of an apical and a basolateral compartment separated by the polarized epithelial cell layer with intact barrier function (reviewed in: Chen and Schoen 2019). In the compartmentalized culture system cells can be grown at the air–liquid interface (ALI; culture medium is only provided in the basolateral compartment) or at the liquid–liquid interface (LLI; cells are additionally covered with a small volume of culture medium from the apical side). Our group successfully optimized compartmentalized culture systems using the ALI approach for long-term culture of OEC of different species including pig, cattle, and mouse (Miessen et al. 2011; Chen et al. 2013b, 2017). In these species, OEC culture at the ALI leads to the formation of functional epithelial tissues in vitro (Chen et al. 2017). The compartmentalized system enables co-culture with embryos/gametes or application of embryonic signals in the apical cell compartment and provision of maternal effectors (e.g. metabolic or hormonal stimuli) in the basolateral compartment. Therefore, such compartmentalized in vitro systems are powerful tools to investigate embryo–maternal interactions and the dynamic composition of the early embryonic environment (Chen and Schoen 2019). The aim of the current study was to apply the compartmentalized culture approach for the establishment of differentiated long-term cultures of FOEC separately isolated from both, the ampulla and isthmus region.

The domestic cat (*Felis catus*) is frequently used as a model species for felids. To prevent undesired pregnancies and control stray populations, cats are routinely neutered in veterinary clinics and animal shelters. Although these cats are highly diverse with respect to age, reproductive and general health status compared to farm and laboratory animals, it is possible to obtain and examine oviduct samples for their use in vitro. We tested whether (i) the compartmentalized culture approach using media optimized for murine and porcine OEC is generally applicable for FOEC, (ii) it is possible to long-term culture cells from the ampulla and isthmus region of the feline oviduct separately and (iii) highly differentiated FOEC cultures can be obtained from the heterogeneous basic material (age, breed, reproductive and health status of the donor animals) available after routine castrations.

## Materials and methods

### Reagents and media

Cell culture media used in culture procedures (Table 1) were modifications of previously described media for mouse tracheal cell culture (You et al. 2002) as well as for ALI culture of murine and porcine OEC (Chen et al. 2017). The cell culture medium DMEM/Ham’s F12 (1:1, FG 415), FBS (S 0115, Lot 1030B) and amphotericin B were obtained from Merck Millipore (Darmstadt, Germany), while other chemicals, media and supplements were purchased from Sigma-Aldrich (Taufkirchen, Germany) unless otherwise stated.

**Table 1:** Media compositions used for compartmentalized cell culture

Media/reagents	FOEC-BASIC	FOEC-PROL	FOEC-DIFF	Transport medium	Washing medium
Basic medium	DMEM/Ham’s F12 (1:1, FG 415)	FOEC-BASIC	FOEC-BASIC	HEPES–MEM (M7278)	PBS (D8537)
HEPES (H0887)	15 mM				
Penicillin, streptomycin (P4333)	100 U/ml, 100 µg/ml				100 U/ml, 100 µg/ml
Amphotericin B (171375)	0.25 µg/ml				0.25 µg/ml
Gentamicin					0.05 mg/ml
Antibiotic antimycotic solution (A5955)				1%	
Insulin (I6634)		5 µg/ml	2.5 µg/ml		
Transferrin (T8158)		5 µg/ml	5 µg/ml		
Cholera toxin (C8052)		0.025 µg/ml	6.26 ng/ml		
Epidermal growth factor (E4127)		5 ng/ml	1 ng/ml		
Bovine pituitary extract (P1476)		30 µg/ml	30 µg/ml		
Retinoic acid (R2625)		0.05 µM	0.05 µM		
FBS (S 0115, Lot 1030B)		5%			
BSA (126579)			1 mg/ml	3 mg/ml	

*PROL*  culture medium used during the proliferation phase, *DIFF* culture medium used for the differentiation phase of the culture process

### Animals

Oviduct tissue samples of 33 domestic cats (*F. catus*) were obtained from the Animal Rescue Shelter or Veterinarians in Berlin, Germany. They were collected by the local veterinarians after routine gonadectomy. The experimental protocols were approved by the Ethics Committee of the Leibniz Institute for Zoo and Wildlife Research (2013-05-05).

### Tissue and sample preparation

Ovaries with oviducts were transported to the laboratory in transport medium at 4 °C and were processed within 5 h. However, the storage time between gonadectomy and transport to the laboratory varied between 1 and 6 h. The organs markedly differed in size and ovarian status. Oviducts from both, late follicular phase ovaries (follicles larger than 2 mm in diameter visible) and inactive ovaries (neither follicles nor *corpora lutea* visible) were used.

Isolation of FOEC was conducted according to a previously published protocol (Eder et al. 2020). Briefly, after removal of the ovary and surrounding tissues, oviducts were washed in washing medium. The oviducts were injected with 1 mg/ml collagenase 1 A (C5894) in PBS (D8537) and incubated for 30 min at 38 °C. Epithelial cells from isthmus and ampulla were squeezed out separately with the outer edge of a scissor onto a glass slide, flushed into 600 µl FOEC-BASIC in a 4-well dish and pre-cultured for 16 h at 38.5 °C, 5% CO_2_. If both oviducts of one cat were available, the two samples of each region were pooled.

During the pre-culture, extracted cell associations formed three-dimensional vesicles. After pre-culture, FOEC-vesicles mainly consisting of viable cells were separated from vesicles comprising many dead cells (Eder et al. 2020). They were resuspended in 10 × trypsin/EDTA (59418 C) solution for subsequent single cell isolation. After 10 min digestion at 38 °C in a water bath, the reaction was stopped by adding 750 µl FBS. Single cell isolation was completed by gentle pipetting through a wide pipet tip and filtering through a cell strainer of 40 μm pore size. Cell concentrations were determined in a counting chamber. FOEC suspensions were centrifuged for 5 min at 200 × g, pellets were resuspended with proliferation medium (FOEC-PROL) and adjusted to a cell concentration of 1 × 10^6^/ml.

### Long-term culture of FOEC

For the long-term culture, 24-well hanging inserts (PET, pore size 0.4 μm, non-transparent, Millipore, Switzerland) were used. Inserts were coated with human placenta collagen (C5533) according to the manufacturer’s instructions.

Depending on the number of isolated viable cells, 1–2 × 10^5^ FOEC were seeded onto the apical side of each insert. Each well (basal side of insert) was filled with 1 ml FOEC-PROL. All samples were maintained submerged during the proliferation phase for 3 to 5 days. Once a confluent cell monolayer was established, cells were either grown at the ALI with 1 ml FOEC-DIFF in the basolateral compartment and without addition of medium on the apical side, or with 1 ml FOEC-DIFF in the basolateral and 50 µl FOEC-DIFF in the apical compartment of the inserts (LLI). Medium in the basolateral compartment (ALI approach) or in both the basolateral and apical compartment (LLI approach) was changed twice a week. During a culture period of 3 weeks the FOEC were incubated in humidified atmosphere with 5% CO_2_ at 38.5 °C.

### Histology and morphological evaluation

**Fig. 1:**
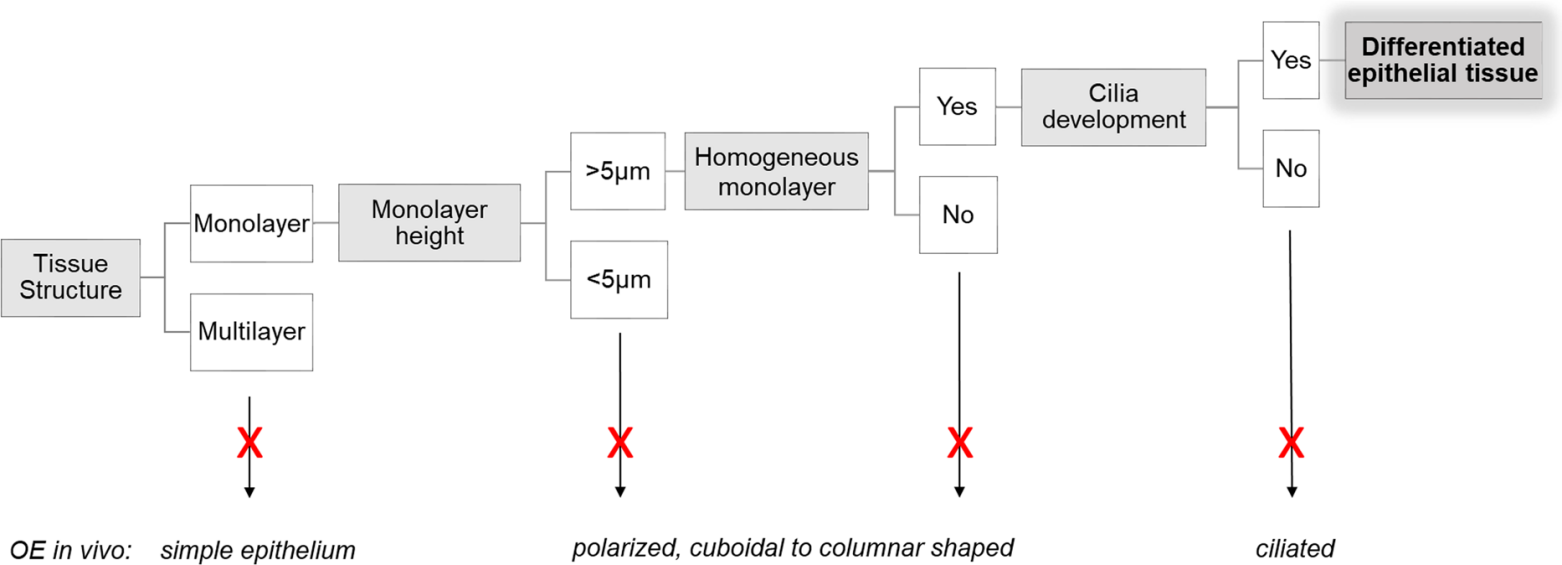
Criteria applied for the morphological evaluation of FOEC after 3 weeks of culture (*OE* oviduct epithelium)

Histological processing was performed as previously described by our group (Chen et al. 2013a, b). Briefly, the cell layer together with the filter support was fixed in Bouin`s solution, stabilized in agarose and post-fixed in 4% phosphate buffered formaldehyde. After dehydration in an ascending ethanol series, the samples were embedded in paraffin. Samples were cut into 3 μm sections, stained with haematoxylin/eosin (HE) and microscopically evaluated for epithelial differentiation using the criteria depicted in Fig. 1.

### Immunohistochemistry

Immunolocalization of marker proteins for cilia development (acetylated tubulin) and oviduct specific secretory activity (oviduct-specific glycoprotein) was conducted in morphologically differentiated cultures. Antigen retrieval was performed either enzymatically (acetylated tubulin) by incubation with 0.06% trypsin, pH 7.8 or via heat-induced antigen retrieval (oviduct-specific glycoprotein) using sodium citrate buffer (10 mM sodium citrate, 0.05% Tween 20, pH 6.0). Unspecific binding sites were blocked with 5% BSA and 10% goat serum in PBS (30 min, room temperature). Slides were incubated with the primary antibodies mouse monoclonal anti-human acetylated tubulin (Sigma T7451; 1:1000 in PBS with 1% BSA) or rabbit polyclonal anti-human oviduct-specific glycoprotein (Abcam ab118590; 1:500 in PBS with 1% BSA), respectively (overnight, 4 °C). The corresponding secondary antibodies were goat anti-mouse IgG, Alexa 568 (Invitrogen A-11031; 1:40 in PBS with 1% BSA) and goat anti-rabbit IgG, Alexa 647 (Invitrogen A-21245; 1:200 in PBS with 1% BSA), respectively, and were applied for 1 h at room temperature. Negative controls were performed by omitting the primary antibody. SYBR Green I (Mobitec, Berkheim) was used for nuclei counterstaining. Pictures were captured using a Zeiss LSM800 equipped with fluorescence optics and ZEN software.

## Results and discussion

The compartmentalized culture approach was originally developed for epidermal and respiratory epithelial cells to better mimic in vivo epithelia (Pruniéras et al. 1983; Whitcutt et al. 1988). Later, this method was adopted for gastric (Tabuchi 2001), ear (Portier et al. 2005), cornea (Sygitowicz et al. 2011) and female reproductive tract epithelia (reviewed in: Chen and Schoen 2019). In cats, only trachea epithelial cells had been cultured using a compartmentalized approach (Nelli et al. 2016).

The oviduct samples that could be obtained from the animal shelter and private veterinary clinics were expectedly very heterogeneous (i.e. obtained from cats of different breeds, ages, and health conditions). Since cats of private owners are often neutered before they come into heat for the first time more samples were available from cats with inactive ovaries. In total, 25 cats with inactive ovaries and 8 cats in the late follicular phase of the estrous cycle were sampled. The cell numbers isolated per oviduct were highly variable (inactive isthmus: 2.1 ± 1.4 × 10^5^, inactive ampulla 4.6 ± 3.8 × 10^5^, active isthmus: 2.4 ± 1.9 × 10^5^, active ampulla: 6.7 ± 4.1 × 10^5^). The reproductive tract of the young and inactive females is still very delicate. Therefore, a minimum of 1 × 10^5^ cells of at least one of the oviduct segments (ampulla or isthmus) could only be collected from 18 individuals with inactive ovaries. This was the case for all eight individuals in late follicular phase. Because of the unbalanced cell numbers per ovary stage, individual and oviduct region, we assigned the original cells in mostly non-paired approaches to the culture treatments. Only five and three “full” sets of sample allocation (isthmus and ampulla each under ALI and LLI condition) were possible in the groups with inactive or active ovaries, respectively. Therefore, we did not perform statistical evaluation and only consider the information on potentially successful culture strategies important for the progress of feline oviduct long-term culture.

**Table 2:** Success of compartmentalized long-term culture of feline oviduct epithelial cells in air–liquid (ALI) or liquid–liquid (LLI) approaches

Ovary stage	Inactive				Follicular phase				∑
Oviduct segment	Isthmus		Ampulla		Isthmus		Ampulla		
Number of segments with ≥ 10^5^ cells	15		18		6		8		
Culture method	ALI	LLI	ALI	LLI	ALI	LLI	ALI	LLI	
Number of cultures with 1–2 × 10^5^ seeded cells	9	9	17	15	5	4	8	4	71
Number of cultures with polarized epithelial monolayers	2	1	0	2	0	0	0	0	5

**Fig. 2:**
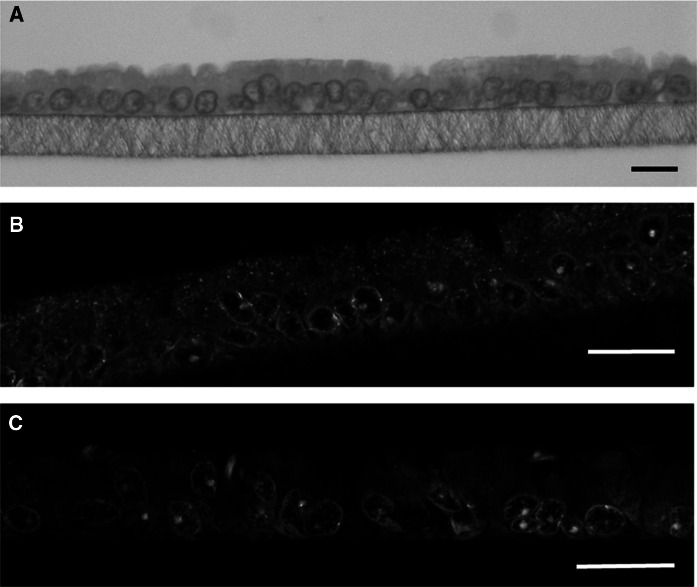
Representative pictures of differentiated feline oviduct epithelial cells after three weeks of culture on permeable filter supports. Cells were obtained from the ampulla of a donor with inactive ovaries and cultured at the LLI. **A** HE staining. Immunofluorescent labeling of oviduct-specific glycoprotein (**B**, red) and acetylated tubulin to visualize cilia (**C**, red). Nuclei in **B** and **C** were stained green by SYBR Green I. Bars represent 10 μm. (Color figure online)

In total, only five cultured samples (all derived from the more numerous females with inactive ovaries) reached a differentiated status representing the simple, cuboidal to columnar shaped oviduct epithelium (Table 2). Three of these samples were obtained from the same donor (isthmus ALI and isthmus LLI, ampulla LLI), further two samples stem from two other females. Differentiated samples formed a homogeneous epithelial monolayer consisting of polarized cells (Fig. 2A) as shown for pig, cattle and mouse by the same approach (Miessen et al. 2011; Chen et al. 2013b, 2017). Oviduct-specific glycoprotein, a marker for functional oviduct epithelia, was expressed in all of the differentiated cultures as exemplary shown for one ampulla LLI sample (Fig. 2B). The presence of cilia was verified by staining of acetylated tubulin. However, cilia formation was a rather rare observation in all five differentiated cultures (Fig. 2C). Prolongation of the differentiation period as previously described for bovine oviduct epithelial cells (Chen et al. 2017) might be necessary to allow in vivo-like cilia formation. Three of the five differentiated cultures were obtained using the LLI and two using the ALI approach. Therefore, both methods seem to be generally applicable. Due to the limited number of successful cultures, however, no conclusions can be drawn as to which condition is more suitable for fostering differentiation.

Our results showed that a long-term differentiated culture of FOEC over 3 weeks is principally possible although the pronounced variability of the native material from routine castrations and the limited cell yield are basic handicaps. To select viable cells, a pre-culture of FOEC-vesicles was applied. However, we suspect that not only the number of viable cells but especially proliferative progenitor-like cells within the isolated cell population is crucial for the success of the compartmentalized culture approach. Previous reports (Kessler et al. 2015) describe the formation of fully differentiated human oviduct epithelium organoids from clonal cells by experimental control of their stemness and, therefore, their proliferative potential prior to induction of differentiation. In species with limited sample availability and heterogeneous sample quality such as human, mouse, cat or wildlife, protocols which increase the proliferative capacity of the primary cells, such as organoid pre-culture to increase the number of progenitor-like cells, are required to accomplish stable, reproducible long-term cultures for application in reproductive research and assisted reproduction.

## Data Availability

The datasets generated during and/or analysed during the current study are available from the corresponding author on reasonable request.
